# Treatment Options in Vulvar Lichen Sclerosus: A Scoping Review

**DOI:** 10.7759/cureus.13527

**Published:** 2021-02-24

**Authors:** Nilanchali Singh, Neha Mishra, Prafull Ghatage

**Affiliations:** 1 Gynecologic Oncology, Tom Baker Cancer Centre, Calgary, CAN; 2 Obstetrics and Gynecology, All India Institute of Medical Sciences, New Delhi, Greater Noida, IND; 3 Obstetrics and Gynecology, Government Institute of Medical Sciences, Greater Noida, IND

**Keywords:** vulvar, vulval, lichen sclerosus, adult, juvenile, treatment, follow-up, review

## Abstract

Vulvar lichen sclerosus (VLS) is a chronic inflammatory disorder, which affects women of all ages. The aim of this review is to focus on first-line, second-line, and maintenance therapies as well as follow-up of women with VLS. With numerous controversies, we decided to conduct a scoping review on this subject. A review protocol was developed, and the Knowledge Resource Services website was used to run a search of articles pertaining to VLS with keywords “Vulvar,” “Vulval,” and “Lichen Sclerosus.” The search was limited to published data from the last 10 years, i.e., July 2009 onward, and researches published in English language. A total of 338 articles pertaining to VLS were obtained. Out of this, 62 were original articles related to management of VLS. Effective treatments such as high-potency topical steroids are now the standard of care and first-line treatment. Follow-up may be done every three to six months for the first two years and then at least yearly to ensure adequacy of treatment and encourage compliance. Long-term follow-up in specialist clinics is recommended for women who have persistent complaints, thickened skin, or history of neoplastic lesion. Monitoring young patients yearly is recommended as there are chances of recurrence.

## Introduction and background

Vulvar lichen sclerosus (VLS), also known as vulvar dystrophy in the past, is one of the most common pathologies presenting to vulvar clinics. A study reported that incidence rate of lichen sclerosus increased from 7.4 to 14.6 per 100,000 woman-years between 1991 to 2011 [1]. The vague terminologies like leukoplakia, kraurosis, and dystrophy of vulva were prevalent before International Society for the Study of Vulvovaginal Disease (ISSVD) 1975 classification system. Presently, this disease is included in non-neoplastic and non-infectious entities; vulvar dermatoses in ISSVD classification includes this disease entity with vulvar dermatoses, which are non-neoplastic and non-infectious in nature [2]. VLS has bimodal presentation, both in pre-pubertal girls and postmenopausal women. Some cases may present in reproductive group (18-40) females. It has multitude of causative factors such as autoimmune pathologies, hormones, and infections. The labia, perineum, and perianal areas get affected and present as a patchy, thin, glistening, and ivory-white area. The clinical features suffice diagnosis, and biopsy is rarely done [3]. As far as the treatment strategy is concerned, testosterone was the mainstay of treatment in the past, whereas high-potency steroids are now considered the standard therapy. Testosterone was found ineffective in root canal treatment (RCTs) and has unacceptable side effects [4]. Follow-up and maintenance therapies are also not clearly defined. With numerous controversies, we decided to conduct a scoping review on this subject. The focus of this review is on the first-line, second-line, and maintenance therapies as well as on the follow-up of women with VLS. Additionally, the focus will also be on the difference in management of adult and juvenile VLS.

## Review

Methods

A review protocol was framed. PubMed was explored for the literature search related to VLS with the assistance of Knowledge Resource Services website. Various databases were searched: MEDLINE (Ovid), Evidence-Based Medicine (EBM) Reviews, PubMed, Cumulative Index to Nursing and Allied Health Literature (CINAHL), MEDLINE, and Excerpta Medica Database (EMBASE). Keywords used for search were: “Vulvar,” “Vulval,” “lichen sclerosus et atrophicus,” “kraurosis,” “dystrophy,” “VIN,” “Cancer,” and “lichen sclerosus.” The published data from July 1999 in English language were accessed. After a comprehensive search, 338 articles concerning to VLS were retrieved. All of these articles were screened; 276 articles were excluded because of the following reasons: not correlated to the objective of this review, i.e., management of VLS, or they were not original articles. This review incorporates 62 studies. Inclusion of original articles and review articles was done on priority basis. In some cases, relevant older data was also included such as role of testosterone in the management of VLS. There are not any papers available regarding testosterone usage owing to its severe unpropitious effects. All the articles were retrieved in full text. This review did not seek individual data sources, and a narrative analysis was done. The data was summarized in the form of a descriptive review.

Therapeutic options for vulvar lichen sclerosus

The various modalities of treatment of VLS are summarized in Table 1.

**Table 1:** Treatment Modalities for Vulvar Lichen Sclerosus

S. No.	Treatment Modalities for Vulvar Lichen Sclerosus
1.	Local Steroid Therapy
2.	Intra-lesional Steroid Therapy
3.	Topical Calcineurin Inhibitors
4.	Topical Androgens, Progesterone, and Estrogens
5.	Other Local Pharmacologic Agents Like Retinoids
6.	Emollients
7.	Phototherapy
8.	Laser Therapy
9.	Photodynamic Therapy
10.	Cryotherapy
11.	Fat Grafting
12.	Adipose-Derived Stem Cells and Platelet-Rich Plasma Therapy
13.	Herbal Therapies
14.	Systemic Therapy
15.	Dietary Modification
16.	Surgical Intervention

1. Steroid Therapy: Local

The gold standard treatment for VLS consists of high-potency topical corticosteroids (TCS), such as clobetasol dipropionate. Clobetasol propionate cream (0.05%) significantly reduces symptoms and improves skin characteristics [5,6]. The most common regimen is to apply it thoroughly over lesion two times daily for three months [7]. As per our experience, we also recommend a constant dosing schedule of twice a day for 12 weeks [5]. Creams are water-based, spread easily, and absorbed easily; however, ointments are oil-based, have less spreadability, and take a longer time to absorb. Ointments are more potent than creams. Creams may contain alcohols or preservatives that are much more likely to cause burning, stinging, and/or irritation, particularly of the inflamed/fissured mucosa in lichen sclerosus, and as such, ointments are recommended over creams as first-line therapy. About 60%-70% of patients experience complete remission of their symptoms [8]. Others, who continue to have flares and remissions after 12 weeks of therapy, are advised to use clobetasol propionate 0.05% as required [9]. The side effects of TCS include irritation, burning, dryness, hypopigmentation, and dermal atrophy [10]. The adverse effects of stinging, burning, and dryness are most commonly due to the base of the topical steroid rather than the steroid itself; hypopigmentation and dermal atrophy may occur with topical steroid use, particularly to keratinized skin, but these side effects are specifically noted to rarely occur in most long-term studies of topical steroids for treatment of VLS [11].

The less-potent steroids like mometasone furoate (0.1%) and triamcinolone are also found to be effective [12]. However, current recommendations favor the initial use of highly potent steroids as first line of therapy with the less-potent steroids used for maintenance.

**Figure 1:**
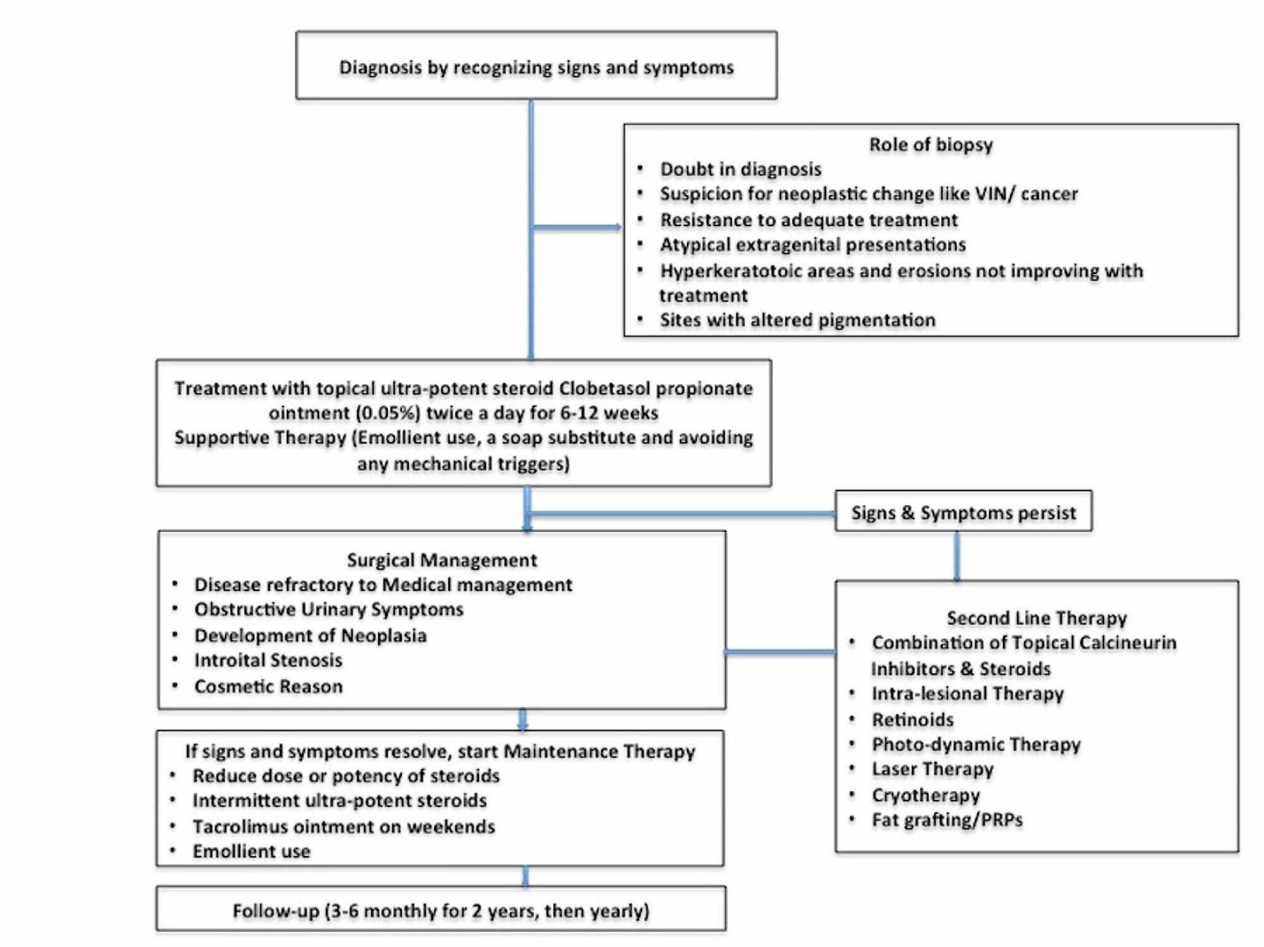
Suggested Management of Adult Vulvar Lichen Sclerosus VIN, Vulvar intraepithelial neoplasia; PRP, platelet-rich plasma.

Some studies have suggested use of different corticosteroids on the basis of severity of disease. They suggest that very severe disease should be treated with clobetasol propionate ointment, moderate to severe disease with betamethasone dipropionate ointment, mild disease with methylprednisolone aceponate ointment, and very mild “burnt out” disease with hydrocortisone ointment [5]. Severity was assessed by the degree of hyperkeratosis of the lesion. Objective return of the vulvar skin to normal color and texture should be the aim to titrate dose and duration of treatment. However, Cochrane Reviews states that potency and dose of topical steroid therapy for treatment of VLS needs further evaluation and research [3]. We therefore recommend what most studies suggest, i.e., usage of clobetasone propionate ointment (0.05%) for treatment of any degree of VLS. The management strategy of adults with VLS is summarized in Figure 1.

2. Intra-lesional Steroid Therapy

Intra-lesional injection of triamcinolone acetonide has been proposed as an alternative treatment to topical treatment of LS, but again the studies are small [13]. There may be a benefit for intra-lesional steroid injection if it thought that there has been a lack of response to a steroid cream or ointment due to poor penetrance. For thick lichen sclerosus, intra-lesional steroid (triamcinolone 3.3-10 mg/ml) may be considered. Pruritic LS has also been treated with TCS or combined corticosteroid and monthly anesthetic/corticosteroid subdermal injection. In a five-year study, time to recurrence was longer with the combined treatment, although women were less satisfied with the injections [14]. Combined treatment can be reserved for women not responding to single therapy. The dose is dependent on the location and thickness of the skin that is being injected, and this can be repeated monthly for two to three months. High steroid doses should not be injected into thin skin or in small areas because the tissue may slough off.

3. Topical Calcineurin Inhibitors

The promising effect of calcineurin inhibitors (CIs) on immunomodulation and in blocking the release of inflammatory cytokines has been used to treat VLS widely. Tacrolimis and pimecrolimus are very effective when it comes to treat anogenital LS topically [15,16]. However, these findings should be taken with a pinch of salt as the studies included a small number of patients [15,16]. They are used as adjunctive therapy for LS but have not been shown to be as effective in initial treatment for LS both clinically and histologically [17,18]. These studies have shown the efficacy of using TCIs daily or twice daily in children and adults with partial to complete remission ranging from six weeks to 10 months of treatment [15,16]. In one study, complete remission was seen in 43% and partial remission in 34% of patients, after using TCIs for 16 weeks [19]. Side effects of TCIs included stinging and burning.

Combining therapy with TCS and TCIs has been studied. An author has reported remission in pediatric cases with LS after alternating clobetasol 0.05% ointment with tacrolimus ointment [20]. Clobetasol 0.05% ointment is used as first-line therapy and tacrolimus ointment as a bridging therapy in between the steroid therapy. As patients improved, clobetasol ointment was discontinued, and tacrolimus ointment was used on weekends as maintenance therapy. Clearance ranged from four to 156 weeks with an average time of 43.1 weeks. TCI use is recommended in women with corticosteroid-resistant disease or intolerance to steroids. The long-term safety profile of TCIs is not established, and there are concerns about the increased risk of neoplasia, especially with long-term use [7].

4. Topical Androgens and Progesterone

Randomized controlled trials have tested and compared testosterone propionate cream (2%), dihydrotestosterone (DHT) cream (2%), and progesterone cream with placebo [21,22]. Two authors applied testosterone for three months and a year, respectively, with no significant differences as compared with placebo [21,22]. A relatively small-sized, randomized placebo-controlled trial DHT for three months found no notable difference. So, topical androgens are not justifiable to use in the treatment of VLS [3]. Similarly, topical progesterone was of no benefit [3].

5. Other Local Pharmacologic Agents

Retinoids have anti-inflammatory properties and reduce hyperkeratinization. Virgili et al. studied topical tretinoin cream (0.025%) in a small group of women with histologically proven LS [23]. With four to 13 months of treatment, symptoms, macroscopy, and histology, all improved. Mild-associated skin reactions are found. Pregnancy is a contraindication for their use owing to teratogenic effects reported by them. Borghi et al. reported that tretinoin 0.025% cream application for six months may represent a useful alternative option to corticosteroids in the treatment of active VLS, acting particularly on hyperkeratosis and pallor [24].

Origoni et al. reported that topical oxatomide has a better relief on itching than placebo due to its anti-inflammatory properties. However, it did not have significant effects on other clinical end-points [25].

Shelley et al. hypothesized infectious etiology in vulvar LS and studied antibiotics. They tested penicillin or cephalosporins in an observational study of 15 women not responding to TCS [26]. Symptoms like pain, pruritus, and burning were relieved. Antibiotics, however, should be used only when there are signs of a secondary infection.

Local application of human fibroblast lysate cream, containing anti-inflammatory cytokines as well as wound-healing growth factors, has been found to be useful in treatment [27].

6. Role of Emollients

Emollients or moisturizers should be an integral part of treatment of VLS. Emollients have been used to soften and protect the skin from cracks in VLS patients. It is also advisable to use emollient cream as a substitute to soap. Application of moisturizers or paraffin prevents skin contact with urine, which may reduce symptoms. Emollients and lubricants can be used during sexual intercourse, which might otherwise be painful. Long-term maintenance therapy with a moisturizing cream can maintain the symptom relief induced by TCS in women with vulvar LS while being safe and inexpensive. This treatment may also be associated with a reduction in topical corticosteroid use with over half of women being able to eliminate the use of corticosteroids altogether [28]. It should be noted that improvement in symptoms allowing for discontinuation of topical steroids may not correspond to reduction in risk of scarring or development of squamous cell carcinomas (SCC) [5].

7. Laser Therapy

Laser therapy has been used in a few small studies for the treatment of VLS refractory to TCS [29,30]. The carbon dioxide laser was used on 40 patients with severe hyperkeratotic VLS not responding to high-potency steroids. Remission was achieved, and their disease was subsequently managed with TCS [29]. The use of fractionally ablative Er:YAG (erbium-doped yttrium aluminum garnet) laser therapy (2940 nm) to a depth of 750 µm was evaluated for recalcitrant VLS. At the last follow-up, more than one year after Er:YAG laser treatment, the patient remained asymptomatic on corticosteroid therapy [30].

There is an ongoing trial on the MonaLisa Touch Laser (DEKA Laser, Florence, Italy) looking at the efficacy and safety of the micro-ablative fractional CO_2_ laser treatment (FxCO2) for VLS (Trial no. NCT03665584). Hopefully, this may throw more light on the role of laser therapy.

8. Phototherapy

Ultraviolet A1 (UV-A1) phototherapy has been used for treatment of VLS. UV-A1 phototherapy with initial irradiation intensity of 24 mW/cm^2^ and a distance of approximately 25 cm showed good results; however, later it is revealed that results were inferior to topical steroid treatment [31]. The UV-A1 doses were intensified during the first five treatment sessions and were administered four times per week for a total of 12 weeks, resulting in 48 irradiations in total. Additional therapy was restricted to the use of emollients. There was significant clinical improvement but was inferior to the current gold standard treatment with topical high-potent corticosteroids. The practicability, relief of itch, and improvement in quality of life are better with corticosteroids. Hence, these options should be reserved for refractory cases and not as first-line therapy.

9. Photodynamic Therapy

Photodynamic therapy (PDT) with the use of topical 10% 5-ALA cream (5-aminolaevulinic acid) has been found to be useful in treating VLS [32]. In a study, after application of freshly prepared 10% 5-ALA cream with a one-cm margin and incubating for three hours, the lesions were irradiated with 100 J/cm^2^ 633 nm red light at 100 mW/cm^2^ [33]. The same PDT procedure was repeated three times at two-week intervals. ALA-PDT is a well-tolerated and effective option for the treatment of VLS. Pain was the main adverse reaction in ALA-PDT. ALA-PDT shows longer remission duration and a higher complete response rate than clobetasol propionate [34]. For patients who relapse after ALA-PDT, steroids can be used as a palliative treatment for those whose symptoms are less severe. If symptoms worsen, ALA-PDT can be administered repeatedly to control recurrent and remaining lesions. Since steroids can alleviate some post-PDT transitory symptoms, a combination of ALA-PDT and corticosteroids might be considered in order to reduce treatment cost [34].

One study reported significant improvement with the use of topical 5-ALA with argon laser light [35]. There is an ongoing trial on the Rivelin® plain patch, which works by forming a protective barrier over a lesion, hence protecting it from further irritation. The premise of the Rivelin® plain patch is a longer pain-free period compared to current treatment with bioadhesive agents, thereby potentially increasing the pain-free period.

10. Cryotherapy

Cryotherapy has been used as a treatment modality for VLS refractory to medical management [36]. Temperature of -186°C under general anesthesia has been used in the past to treat VLS. Contact method with one freeze-thaw cycle per lesion and session combined with intra-lesional steroid injection (triamcinolone acetonide) has been used successfully [13]. Postoperative wound care can be done with emollients, compresses, and antiseptic solution. It has been used successfully in children with good acceptance [36]. Cryotherapy may be used as a second-line therapy option in the treatment of long-standing VLS resistant to treatment. However, limiting factors of cryotherapy are a relatively high number of relapses, a long postoperative healing time, and a long time for pain-free status after cryotherapy.

11. Fat Grafting

Fat grafting may be considered a supportive or second-line therapy. It may be used in selected cases of VLS not responding to first-line therapy or for anatomical correction to improve sexual function and quality of life [37]. The rationale of this regenerative approach is the presence of stem cell progenitors within adipose tissue capable of differentiating into different mesenchymal tissues and producing anti-inflammatory and immunomodulatory effects. The results of fat grafting are promising; however, further studies are required [38].

12. Role of Adipose-Derived Stem Cells and Platelet-Rich Plasma Therapy

Adipose-derived stem cells, now considered as stem cell transplant, are purported to be able to restore and regenerate damaged tissue as in VLS. They have tissue regenerative potential in fibrotic conditions. Adipose-derived stem cells proliferate and differentiate into various mesenchymal tissues. They also have anti-inflammatory and immunomodulatory properties. These factors are hypothesized to ultimately inhibit fibrosis, promote healing, and remodel the extracellular matrix. Similarly, platelet-rich plasma (PRP) therapy acts as a regenerative agent as it has tissue factors for cell growth, migration, repair, proliferation, and regeneration. It is still in experimental stages with few reports of successful management of patients with VLS [39]. Prospective randomized trials are needed to provide an evidence base for this therapy.

13. Herbal Therapies

Medicinal plants with their metabolites have been used as a complementary medicine in various inflammatory diseases. Herbal therapies with agents like chamomile, coconut oil, shea butter, aloe vera, calendula, arnica, avocado, etc. [40] have been used in VLS with variable results. The fact that whether they act as emollient or have any therapeutic potential is controversial. Further studies are required to prove their efficacy in treatment of VLS.

14. Systemic Therapy

If pruritus persists after corticosteroid and other local therapies, hydroxyzine or doxepin may be given to stop nighttime pruritus. In cases of worse pruritus, subcutaneous triamcinolone in the vulvar tissue has been attempted [41]. Acitretin is a retinoid that may be used for lichen sclerosus unresponsive to topical steroids [42]. It is most beneficial for thickened skin due to vulvar sclerosus, which is unresponsive to topical agents. It should not be prescribed if the patient is pregnant or planning for the same, as it is teratogenic.

15. Role of Dietary Modification

There are some reports of effect of diet on disease symptomatology and progression of VLS [43]. Pork consumption has been associated with worsening of symptoms in VLS [44]. Other foods advised to be avoided in VLS are spinach, canned and boxed food items, potatoes, nuts, cocoa, beets, turnip, etc. [44]. These items are high in oxalate, which leads to worsening of symptoms in VLS. However, there is no clear evidence for association of these dietary habits with VLS. Some clinicians advocate calcium-rich diet.

16. Surgical Intervention

Surgical management of labial agglutination in VLS should be reserved for patients with disease refractory to medical management or with obstructive urinary symptoms. Lysis of adhesion may be required for labial fusions and clitoral hood scarring for cosmetic reasons too. Surgical separation of labial agglutination in postmenopausal women can result in recurrence of labial adhesions and scarring of the tissue. Refractory or recurrent labial agglutination in postmenopausal women can result in symptomatic architectural changes of the vulvovaginal anatomy. In severe introital stenosis, labial lysis of adhesion may not be adequate, and more extensive procedures such as Fenton's operation of a longitudinal incision through the posterior fourchette and perineal body may be required. Surgical separation of tissue can often result in adhesions and increased scarring of the tissue due to recurrence of lichen sclerosus at sites of tissue injury, known as the Koebner phenomenon. Surgical treatment of clitoral phimosis can improve sexual function [45]. Vulval LS recalcitrant to medical treatment or that recurs following simple excision or adhesiolysis may successfully be treated with wider excision and reconstruction with split skin graft (SSG). Oxidized regenerated cellulose has also been studied to prevent recurrence of adhesions [46]. More aggressive surgical treatment like vulvectomy is reserved for women with malignancy or post-inflammatory sequelae [47]. More studies are required as far as surgical management of VLS is concerned.

17. Maintenance Therapy

Once the VLS lesion has responded to treatment, maintenance therapy is often required as a preventive measure. Normalcy of skin color and texture is the target, and the dose of topical steroids may be titrated depending upon the outcome. Dosing therapy may vary from thrice a week to daily depending upon the severity. Some advises an intermittent highly active topical steroid agent as and when required [5]. Others believe that patients with moderate to severe lesion may require a less-potent steroid therapy or topical CIs for a long time till complete remission [48]. Individualized preventive TCS regimens used by compliant patients modify the course of the disease. There is a significant difference in symptom control, scarring, and occurrence of vulvar carcinoma between compliant and partially compliant patients [5]. If side effects like atrophy or corticosteroid dermatitis develop, the potency of the TCS should be reduced. If, however, hyperkeratosis returns, the potency of the TCS should be increased. Therefore, there is no fixed dosage regimen that can be followed for maintenance therapy. Long-term maintenance therapy with a moisturizing cream can maintain symptom relief induced by TCS while being safe and inexpensive. Emollient use may also be associated with a reduction in topical corticosteroid use. If one is not responding to treatment, other conditions should be investigated and ruled out, and biopsy should be considered.

18. Treatment of Juvenile Lichen Sclerosus

**Figure 2:**
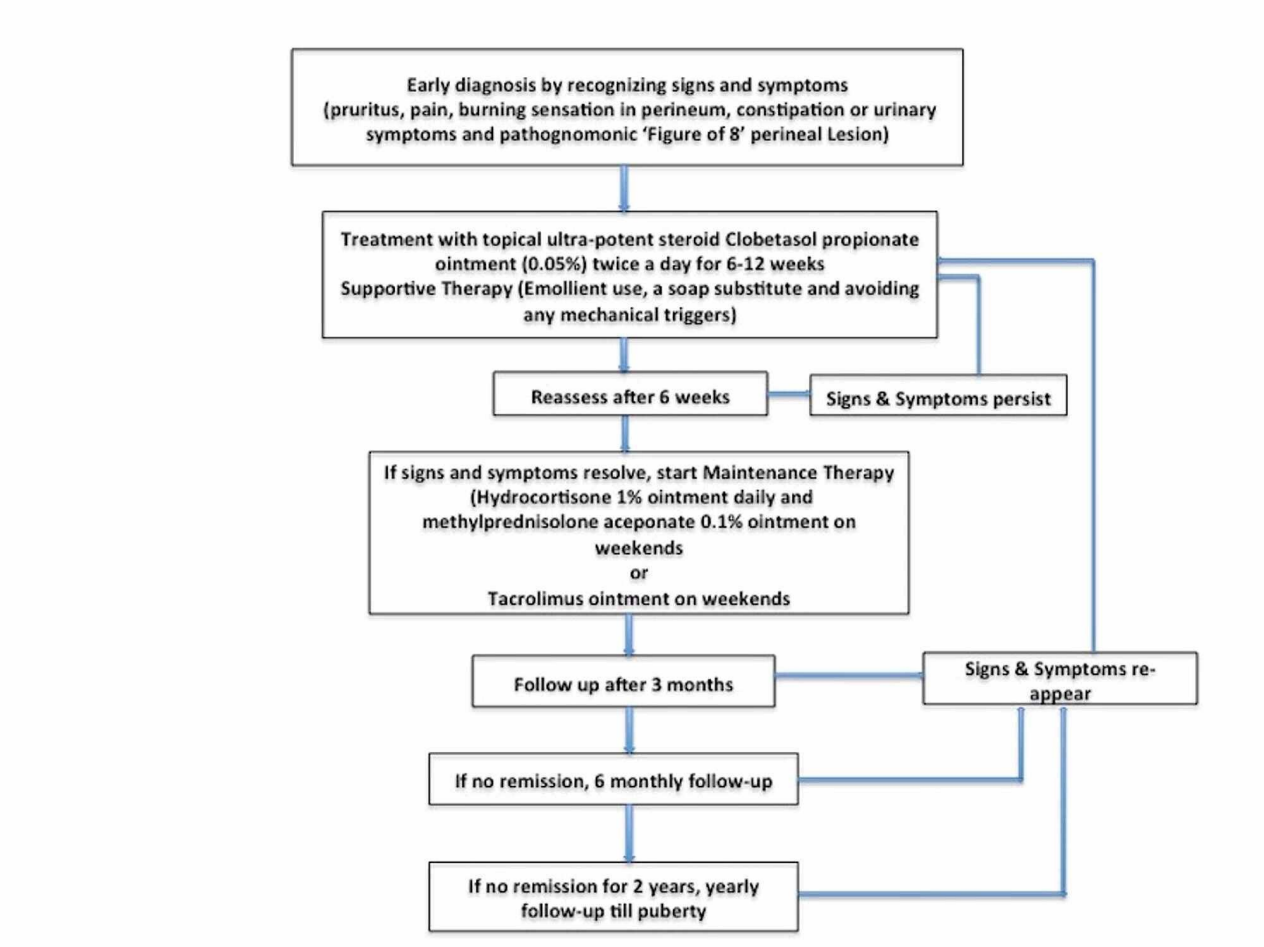
Suggested Management of Pediatric Vulvar Lichen Sclerosus

Topical steroids are the mainstay of treatment, just like their adult counterpart. Potent or very potent topical steroids, applied once or twice a day for six to 12 weeks, with progressive weaning, are the treatment of choice. In a case series of pediatric LS, successful treatment was obtained with TCS (betamethasone dipropionate 0.05% ointment, diflorasone diacetate 0.05% ointment, or clobetasol propionate 0.05% ointment) twice daily for six to 8 weeks. Minimal side effects were observed [49]. While mid-potency TCS, such as triamcinolone acetonide or mometasone furoate, have been found to be effective in pediatric LS, most current recommendations do not support their use for first-line treatment. Supportive therapy is in the form of emollient use and a soap substitute. If symptoms resolve, TCS potency can be gradually reduced, and maintenance therapy can be started. A combination therapy including hydrocortisone 1% ointment and methylprednisolone aceponate 0.1% ointment is recommended for maintenance. Low-concentration topical tacrolimus has been shown to be an effective and safe treatment for children with anogenital lichen sclerosus [50]. VLS usually improves at puberty but can persist into adulthood. Monitoring the patients yearly is recommended as there are chances of recurrence. The management strategy of juvenile VLS is summarized in Figure 2.

Patient counseling and vulvar hygiene

Patient should be counseled to avoid using soap and use emollients instead. Drying of skin after passing urine to prevent its contact is useful. Using a moisturizer or paraffin as a barrier cream is protective against exposure to urine. Lubricants and, if required, vaginal dilators may aid if intercourse is painful, which may happen with VLS. Light, soft, airy, and frictionless materials for undergarments like Derma silk etc. have been shown to improve the symptoms and are recommended. Patients should be counseled regarding the risk of developing vulvar cancer and, therefore, the need of self-examination to notice any change in skin color, texture, or development of ulcers etc. Asymptomatic LS should also be treated in an attempt to decrease the risk of progression to vulvar SCC(7). Women should be encouraged to examine their vulva for any changes, though this may be difficult in older women with co-morbidities such as osteoarthritis, visual problems, and obesity. Patients should be advised against smoking as it increases the risk of vulvar cancer.

It is important to emphasize that LS needs to be treated even if asymptomatic to prevent scarring leading to sexual and urinary problems. While treating, we need to comment that follow-up should initially be every three months to assess response - decrease in itching and pain. One needs to ensure that there is no atrophy of the skin because of treatment with steroids.

Follow-up, prognosis, and recurrence

Regular follow-up is necessary as there is an increased risk of developing SCC. However, there is no consensus between frequency and duration of follow-up. Follow-up may be done every three to six months for the first two years and then at least yearly to ensure adequacy of treatment and encourage compliance [5].

The features that need to be looked upon on follow-up visits includes compliance of treatment, symptomatic response, objective clinical response to treatment, need of maintenance therapy, development of adhesions or scarring, adverse effects of treatment, and development of SCC or vulvar intraepithelial neoplasia [5]. Long-term follow-up in specialist clinics is recommended for women who have persistent complaints, thickened skin, or history of neoplastic lesion.

## Conclusions

VLS is a chronic inflammatory disorder, which affects women of all ages. Highly potent topical corticosteroids are the first-line treatment. However, their long-term use includes skin thinning. Side effects with local steroids are rare, and it is usually a well-tolerated therapy. Most of the second-line therapies are in experimental phases, and further data is required. Follow-up in these women is essential as there is a likely risk of malignant transformation in around 4% women.
